# Safety and Feasibility of Robot-assisted Gait Training in Adults with Cerebral Palsy in an Inpatient Setting – an Observational Study

**DOI:** 10.1007/s10882-023-09895-8

**Published:** 2023-02-11

**Authors:** Fabian Moll, Axel Kessel, Anna Bonetto, Johanna Stresow, Monika Herten, Marcel Dudda, Jens Adermann

**Affiliations:** 1 Klinik für Manuelle Therapie Hamm (Hospital for Pain Management), Ostenalle 83, 59071 Hamm, Germany; 2 Department for Trauma-, Hand- and Reconstructive Surgery, University Hospital Essen, University of Duisburg-Essen, Hufelandstraße 55, 45147 Essen, Germany; 3 Hufelandstraße 55, 45147 Essen, Germany

**Keywords:** gait disorders neurologic; pediatrics; walk test; robot-assisted gait training;, hybrid assistive limb;, cerebral palsy;, exoskeleton device;, task-specific training

## Abstract

Background: To investigate the safety and feasibility of six sessions of Hybrid Assistive Limb (HAL) robot-assisted gait training (RAGT) integrated into an inpatient therapy concept and their influence on walking speed and gait parameters in adult CP patients. Methods: Eleven subjects (male = 8, female = 3, mean age: 23 years and 2 months, ± 4.5 years) with spastic CP underwent six 20-minute RAGT sessions with the HAL during an 11-day hospital stay. Additionally, physiotherapy, physician-performed manual medicine, massage and exercise therapy were provided. Pre- (T1) and post- (T2) intervention assessments were: 10-metre walking test (10MWT), 6-minute walking test (6MWT), Gross Motor Function Measure (GMFM-88) and lower extremities passive range of motion (pROM). Results: All subjects completed the study. No adverse events were noted. Walking speed in the 10MWT test increased from 32.5 s (± 24.5 s) at T1 to 27.5 s (± 21.4 s) at T2, without significance. Slight, but non-significant improvements were detected in the 6MWT, GMFM and pROM. Confounding factors did not significantly affect the results. Conclusion: Intensive therapy including HAL training leads to non-significant improvements. Further studies with more patients and longer intervention time could provide further insights into the RAGT therapy of adult patients with CP. Registration DRKS-ID: DRKS00020275.

## Introduction

Cerebral palsy (CP) is one of the most common and most expensive paediatric chronic movement disorders (Oskoui et al., 2013). The estimated lifetime cost per patient with CP in the USA and Europe amounts to as much as $ 921,000 (Shih et al., 2018; Oskoui et al., 2013) report a prevalence of 2.1/1000 live births in their meta-analysis, and in some countries incidences of up to 3.2 cases per 1000 births have been recorded (Johnson, 2002; McGuire et al., 2019). Management of patients with CP includes a detailed assessment of motor skills, provision of positioning and mobility aids and health information (Graham et al., 2016). Activity-based therapy approaches such as physiotherapy are the treatment options which have been most intensively studied and shown most evidence of success (Damiano, 2006; Novak et al., 2013). Since the early 2000s, the range of therapies has been supplemented by various forms of robot-assisted gait training (RAGT) (Rosen and Ferguson, 2020) which provide more contextual and sustainable support to patients and improve functional therapy outcomes.

Since the early 2000s various exoskeletons were developed to guide and support walking (Rodríguez-Fernández et al., 2021). Up to now research on the use of RAGT for patients with CP shows that this method has positive effects on different walking parameters and motor functions, with significant changes in gait speed, mean step length or cadence (Bunge et al., 2021). The Hybrid Assistive Limb (HAL) seems to be the only RAGT using electromyographic (EMG) biofeedback to interact with the patient using it, making the HAL unique. Most of the studies using the HAL reported on single cases or case series with different numbers of interventions (1 to 16 training sessions) and patients’ characteristics. Quality assessed with the critical McMaster appraisal tool showed scores from 36.3 to 72.7% (Bunge et al., 2021). The targeted GMFCS levels range from level I to IV (n = 62) with the majority of subjects classified with level II and III (n = 38), mainly diagnosed with spastic diplegic CP (n = 39) and a mean age from 8.5 to 18.2 years (Bunge et al., 2021). No studies using the HAL specified the subjects daily abilities or need for care. Matsuda et al. (2018a, 2018b), Takahashi et al. (2018) and Ueno et al. (2019) included children but adults with CP as well in their studies. Only Nakagawa et al. (2019) reported in their case study mean Borg Scale values of 11.9 (range 11–13) in twelve sessions of HAL training (n = 1) to determine the subjects physical effort. Studies with other exoskeletons without EMG biofeedback on patients with CP using i.e. the Lokomat only focus on children with CP and do not find any statistically significant changes in outcome variables such as walking speed or step length (Olmos-Gómez et al., 2021).

Studies on adult patients after spinal cord injury have also showed promising results after the use of RAGT regarding functional (Jansen et al., 2017) and neuroplastic aspects (Sczesny-Kaiser et al., 2015). However, although there is some literature showing a positive effect of RAGT on people with CP generally, no study has so far focused specifically on adults with CP.

The aim of the study was to investigate whether RAGT is safe and feasible for adults with CP during an 11-day hospital stay (GMFCS level II and III), and furthermore, if six sessions of HAL gait training integrated into an inpatient therapy concept have a significant influence on walking speed and gait parameters in adults with CP. We hypothesize that RAGT with HAL during a 11-day therapy inpatient stay can be delivered feasible and safe in adult patients with CP. It might change results walking test, such as 10-metre walking test (10MWT), in short term.

## Participants and Method

### Study Design

This study is a single group, single centre observational study assessing pre- and post-intervention results. The study protocol was approved by the ethics committee of the Westphalian Wilhelms University of Münster, Germany (2019-581-f-S) and was conducted according to the Declaration of Helsinki. The study protocol was explained to the subjects and parents by a physiotherapist and a physician and illustrated by videos (Klinik für Manuelle Therapie (Hamm), 2020a; Klinik für Manuelle Therapie (Hamm)), 2020b). Written informed consent was obtained before the intervention was carried out. The study was conducted in accordance to the Declaration of Helsinki at the Klinik for Manuelle Therapie, Hamm, Germany (KMT).

### Recruitment and Blinding

Recruitment consecutively took place in the tetraparesis department of the KMT as a sample of convenience. Due to the availability of the HAL the recruitment was performed from May 2021 to December 2021. The inclusion and exclusion criteria were based on existing literature and the mechanical properties of the HAL. Table 1 gives an overview of the inclusion and exclusion criteria of the study. All participants were recruited from the patient population from the clinic. Blinding of the therapist or participants due to the kind of intervention was not possible. The data were analysed anonymously.

**Table 1:** Inclusion and exclusion criteria

Inclusion criteria	Exclusion criteria
• Spastic Cerebral Palsy	• Lower limb surgery in the past 6 months
• Age over 18 years	• Botulinum toxin therapy in the past 3 months
• GMFCS III + II	• Use of alternative RAGT in the past 6 months
• 14 m walking ability	• Current fractures or skin lesions
• Adequate pain reporting (CFCS level I – III)	• Impaired peripheral blood supply
• Ability to follow instructions (CFCS level I – III)	• Inability to follow instructions
• Written informed consent	• Height under 150 cm and over 170 cm

GMFCS: Gross Motor Functions Classification System; RAGT: Robot−assisted gait training; CFCS: Communication Function Classification System

### Study Protocol

**Fig. 1:**
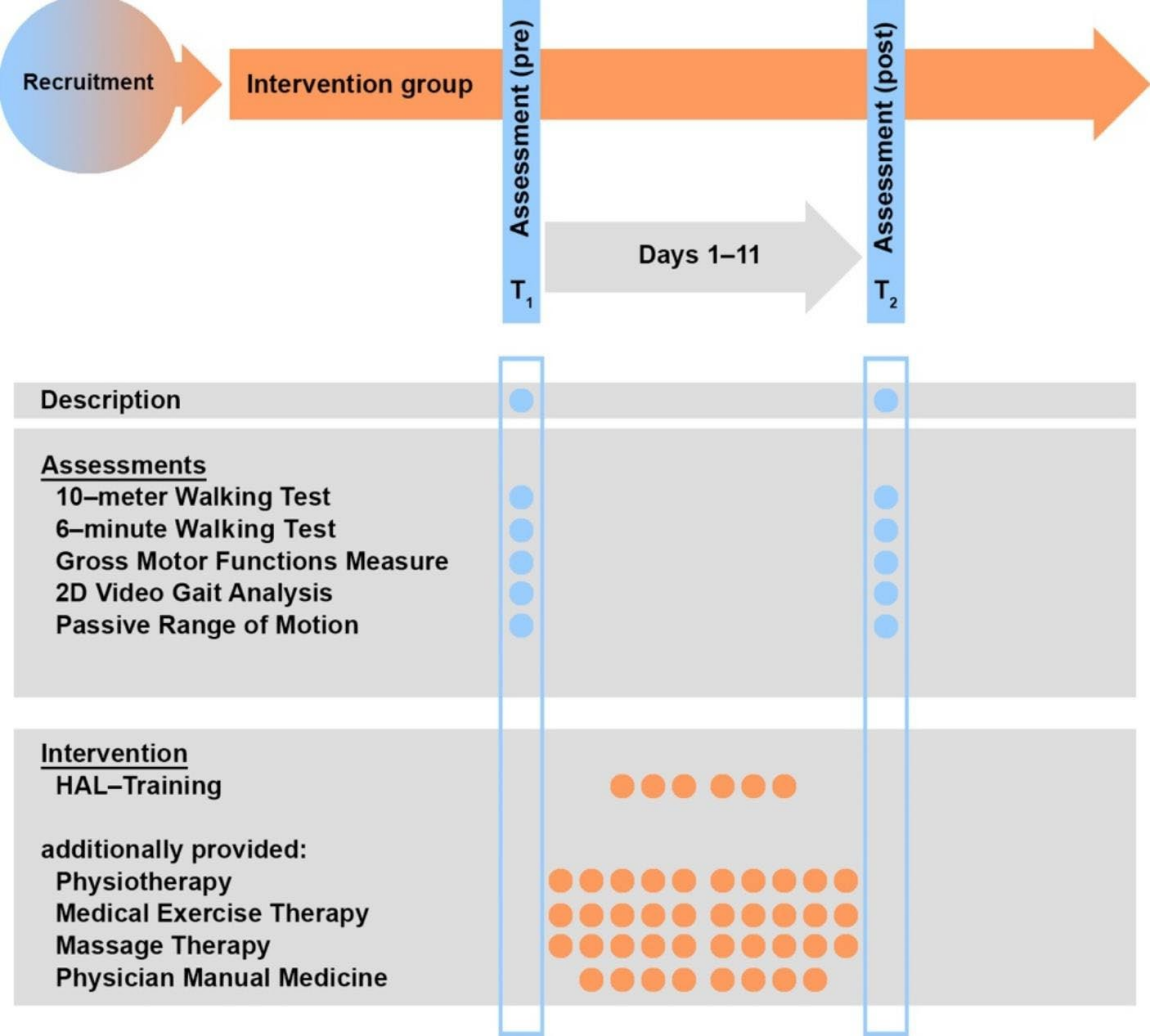
Study design. GMFM-88: Gross Motor Function Measure (88 tasks); Illustration modified from Ammann-Reiffer et al., (2017)

Participants participated in the study during an inpatient stay of 11 days. The participants received the standard therapy concept of the KMT and, additionally, six sessions of RAGT with the HAL (Fig. 1).

### Hybrid Assistive Limb

The HAL (lower limb type ML-05, Cybderdyne Inc., Tsukuba, Japan) is a 14 kg rigid exoskeleton enabling individual joint support by electric motors. Electromyogram skin surface electrodes take the lower extremity muscles’ information and the HAL transforms them into robotic joint movement at the level of the knee and hip joints on sagittal plane (Kawamoto & Sankai, 2005). Interactive biofeedback (iBF) is used to graphically display the leg muscles’ derivations and range of motion to the participant and physical therapist. The RAGT took place in an inpatient setting on a treadmill (Kardiomed Mill, Proxomed Medizintechnik GmbH, Alzenau, Germany) using an overhead lift (HM 2815LRC, Handi-Move International, Ninove, Belgium). The HAL was used in cybernetic control mode with individual settings for each participant.

### Therapy Concept (Intervention)

The robot-assisted gait training with the HAL was carried out for all participants by one physiotherapist who was specially trained by Cyberdyne Inc. prior to the study performance. For each participant was six sessions of gait training with the HAL each 90 min were planned. Each training session includes the actual walking time in the HAL (20 min), time for putting on and taking off the HAL, rests and evaluation adverse events. In addition to the HAL training during the 11-day inpatient clinic stay, the participants also participated in the clinic’s therapy concept presented in Table 2. The therapy focused primarily on individual movement restrictions, pain modalities and functional abilities with the goal of achieving patient participation.

**Table 2:** List of interventions carried out in the inpatient therapy concept according to the Template for Intervention Description and Replication (TIDieR) Checklist and Guide

Intervention	Why?	Who?	How much?
DocManMed	Treatment of local movement disorders; pain modulation; education of patients and attendants	Physician with postgraduate training in manual medicine	4x/ week, 30 min
PhysioNeuro	Facilitation of movements and movement transitions; education; improvement of coordination; promotion of independent and directed movements; increase of concentration and reception skills; promotion of participation.	Physiotherapist with postgraduate training in physiotherapy on a neurophysiological basis	5x/ week, 30 min
PhysioMT	Treatment of local movement disorders; education; pain modulation	Physiotherapist with postgraduate training in musculoskeletal therapy	
MET	Development of functional strength; training control; coordination training; promotion of independent and directed movements; promotion of participation.	Physiotherapist with postgraduate training in medical exercise therapy	5x/ week, 30 min
MassTher	Regulation of muscle tone; pain modulation; physical and mental relaxation; local blood circulation stimulation	Massage therapist	5x/ week, 30 min
HAL	Facilitation of movement transitions; coordination and harmonization of standing leg to swing leg phase transition; postural alignment; contextual gait training; support and completion of movement to promote proprioception.	Specially trained physiotherapist	5x/ week, 30 min

PhysioNeuro: Physiotherapy on a neurophysiological basis, PhysioMT: Musculoskeletal Therapy (Manual therapy); DocManMed: Physician−performed manual medicine; MET: Medical exercise therapy; HAL: Hybrid Assistive Limb; MassTher: Massage therapy. All interventions were performed face−to−face at the KMT Hamm over an 11−day inpatient hospital stay

### Outcome Measures

The functional assessments were performed at day 1 (T1) and day 11 (T2) of the 11-day clinic stay. Primary outcome measure was the 10-metre walking test (10MWT) with self-selected walking speed (SSW). Secondary outcome measures were the 10MWT with maximum walking speed (max.). Gross Motor Function Measure (GMFM total and dimensions standing [D], walking, running and jumping [E]), 6-minute walking test (6MWT), Pedoscan and passive range of motion (pROM) of the lower extremities.

### Statistical Analysis

Descriptive analyses were done to summarize socio-demographic, clinical and gross motor characteristics. Normal distributions of the outcome variables were evaluated based on visual observation of histograms. We compared baseline values of walking and gross motor parameters by a multivariate analysis of covariances (MANCOVA) with repeated measures “within” factors. Confounding variables were selected based on the socio-demographic, clinical and gross motor characteristics. As the RAGT was added to a standard therapy concept of the KMT, a interim analysis or stopping guidelines were planned. A total of 18 subjects would be sufficient to detect an 0.05 m/s significant increase of walking speed in 10MWT (SSW) with an 80% power. This sample size and power was calculated using the software G*Power (version 3.1, Fraul, Erdfelder, Lang, and Buchner, Kiel, Schleswig-Holstein, Germany). A level of *p* < 0.05 was used to indicate statistical significance. All data were analysed using the Statistical Package for Social Sciences (SPSS) version 28 (IBM Corp., Armonk, NY, USA). Adverse events were graded according to Common Terminology Criteria for Adverse Events version 5.0. The minimal important difference (MCID) in 10MWT in adults with different diagnoses, such as spinal cord injury, stroke and traumatic brain injury, is described with 0.05–0.14 m/s (Watson, 2002; Perera et al., 2006; Musselman, 2007). MCID in 6MWT range from 34.4 to 50 m in geriatric patients and subjects with stroke (Perera et al., 2006; Tang et al., 2012). Gross Motor Functions Measurement (GMFM-88) is indicated to have a total score MCID of 0.1–3.0% in children with CP (Storm et al., 2020). No data is available concerning adults with CP.

## Results

Eleven (male = 8; female = 3) participants were recruited consecutively from the tetraparesis department of the hospital. Mean age of 23 years and 2 months (± 4.5 years, range 18–33 years). Average height was 162.4 cm (± 4.5 cm; range 152–168 cm), mean BMI was 23.9 kg/m^2^ (± 4.6; range 16.5–32.2 kg/m^2^). All subjects were classified according to Communication Function Classification System as level I and according to the GMFCS as GMFCS III (n = 10) and GMFCS II (n = 1). Current medication only was baclofen (n = 2). All participants used a wheelchair and various additional aids: 4-point walking stick (n = 2), 1-point walking sticks (n = 1), retro-walker (n = 2), forearm crutches (n = 1), 4-point walking sticks and a retro-walker (n = 4) and 1-point walking sticks and a rollator (n = 1). Table 3 gives summarizes the participants’ characteristics.

**Table 3:** Particpants’ characteristics

Subject	Age	Sex	Height	Weight	BMI	GMFCS	Barthel	Paresis	Orthosis	Therapy
1	19	M	163	68	25.6	3	70	Tetra	AFO	PT, OT
2	27	F	161	71	27.4	2	70	Tetra	AFO	PT, LO
3	23	F	158	52	20.8	3	90	Tetra	none	PT, OT
4	20	M	168	77	27.3	3	65	Tetra	AFO	PT, OT
5	33	M	165	45	16.5	3	75	Tetra	AO	PT, OT
6	22	M	167	67	24.0	3	65	Tetra	AFO	PT
7	18	M	162	77	29.3	3	65	Tetra	Soles	PT, HIP
8	26	M	165	53	19.5	3	25	Tetra	Shoes	PT
9	26	M	164	84	31.2	3	50	Tetra	AFO	PT, OT
10	22	M	152	48	20.8	3	60	Tetra	Shoes	PT
11	19	F	161	53	20.4	3	65	Tetra	AFO	PT

GMFCS: Gross Motor Functions Classification System; M: Male; F: Female; AFO: Ankle−foot orthoses; AO: Ankle orthoses; L: Left; R: Right; PT: Physiotherapy; OT: Occupational therapy; HIP: Hippotherapy; LO: Speech therapy; Tetra: Tetraparesis; Soles: Insoles; Shoes: Custom−made orthopaedic shoes; Barthel: Barthel Index

### Feasibility and Safety

All participants were able to complete the study without any undesired adverse events, such as skin lesions, fatigue, or pain. The HAL could be attached in all subjects easily by a specially trained physical therapist. Attachment of the HAL took 15–20 min depending on the GMFCS level and the individual motor abilities of a participant. Bodyweight support during walking on the treadmill was needed in all participants. In the progress of the RAGT four patients were able to walk free without handrail for a short time. All participants were able to feel the individual support of the HAL during standing and walking. They all made positive comments on the feeling of having no gap between initiation of the movement and support the by the HAL. All participants felt supported and facilitated by using the HAL. As participants with visual limitations, e.g., 13.0 diopters, have severe problems in limb coordination and recognizing the different indicating lines of iBF on the HAL monitor or laptop screen, they expressed that the clear movement input from the HAL is helping them to move more appropriate. Overall, all participants were able to explore coordination and harmonization of lower limb movements on sagittal plane and felt it’s impact on postural alignment during walking and standing.

All subjects performed all six RAGT sessions with the HAL. The average total walking distance in the HAL in all six sessions was 895.3 metres (± 604.1 metres; range 136.7–2054.2 metres). With mixed feelings of motivation and respect using the HAL as a walking aid first time, all participants grow familiar with the HAL in the first two RAGT sessions. The main aspects getting used to the HAL were the participant’ control over the robot support, a calm and safe therapy setting and a good relationship to the serving physical therapist. All participants increased their walking distance in six sessions of RAGT with the HAL in the course of the training sessions.

Evaluation of the data was performed via histograms and showed normal distribution. For the analysis of the functional assessments, a MANCOVA with repeated measurements was calculated with 10MWT (SSW), 10MWT (max), the 6MWT, the GMFM (total) and the GMFM (D + E). There were no significant changes from T1 to T2 across the main effect time within-subjects (F (5,1) = 25.982, p = 0.148; partial η^2^ = 0.992, n = 11). For the included confounding variables age, gender, weight, height and Barthel Index value in interaction with the main factor time, no significant influence on the change in the results of the above-mentioned functional assessments could be reported either. Table 4 gives an overview of the inner subject contrasts of the dependent variables. Due to the low number of participants the study is underpowered.

**Table 4:** Overview of the MANCOVA results and the interaction effects of the interactions of the main factor time with the dependent variables

Measure (time)	F-Value	p-Value	Partial η^2^
10MWT (SSW)	F = (1,5) 0,006	0,942	0,001
10MWT (max.)	F = (1,5) 0,010	0,925	0,002
6MWT	F = (1,5) 0,167	0,700	0,032
GMFM (total)	F = (1,5) 3,388	0,125	0,404
GMFM (D + E)	F = (1,5) 4,954	0,077	0,498

10MWT: 10-metre walking test; 6MWT: 6-minute walking test; GMFM: Gross Motor Function Measure; SSW: Self-selected walking speed; max: Maximum walking speed; D + E: Dimension D and E; p < 0.05; Confidence interval: 95%; n = 11.

**Fig. 2:**
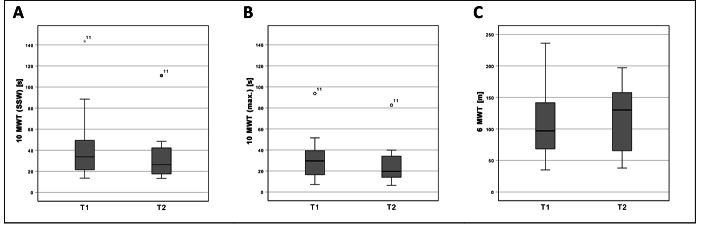
Results of the walking tests. 10-metre walking test with SSW (A) and maximum walking speed (B) and results of the 6-minute walking test (C). Boxplots: Middle line: median; lower whisker: minimum value; upper whisker: maximum value; circles: Outliers (1.5 times interquartile range); SSW: Self-Selected Walking Speed; T1: measurement pre-intervention; T2: measurement post-intervention; p < 0.05; Confidence interval: 95%; n = 11.

The time required in the 10MWT (SSW) changed from 46.0 ± 38.8 s (MW ± SD) (T1) to 35.7 ± 28.1 s (T2) (F = (1,5) 0,006; *p* = 0,942) (Fig. 2). As covariates age, sex, weight, height and Barthel Index value were included in the statistical model.

**Fig. 3:**
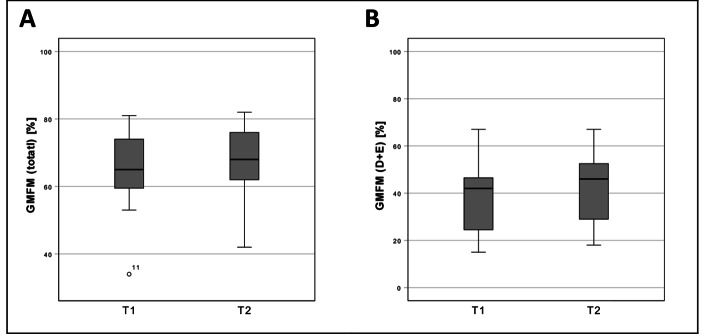
Results of the Gross Motor Function Measure. GMFM in total (A) and Dimension D + E (B). Boxplots: Middle line: median; lower whisker: minimum value; upper whisker: maximum value; circles: Outliers (1.5 times interquartile range); T1: measurement pre-intervention; T2: measurement post-intervention; p < 0.05; Confidence interval: 95%; n = 11.

### Secondary Outcome

#### 10-metre Walking Test (max.)

The time required in the 10MWT (max.) changed from 32.5 ± 24.5 s (T1) to 27.5 ± 21.4 s (T2) (F = (1,5) 0,010; *p* = 0,925) (Fig. 2). As covariates age, sex, weight, height and Barthel Index value were included in the statistical model.

#### 6-minute Walking Test

The distance walked in the 6MWT increased from 111.1 ± 60.8 m (T1) to 115.5 ± 58.1 m (T2) (F = (1,5) 0,167; *p* = 0,700) (Fig. 2). As covariates age, sex, weight, height and Barthel Index value were included in the statistical model.

#### Gross Motor Function Measure

The GMFM (total) scores increased from 64.9 ± 13.5% (T1) to 67.6 ± 12.0% (T2) (F = (1,5) 3,388; *p* = 0,125). The GMFM (D + E) scores increased from 38.8 ± 16.8% (T1) to 42.4 ± 16.7% (T2) (F = (1,5) 4,954; *p* = 0,077) (Fig. 3). As covariates age, sex, weight, height and Barthel Index value were included in the statistical model.

### Passive Range of Motion of the Lower Extremities

A multivariate analysis of variance was not possible due to insufficient degrees of freedom of the residuals. The results of the passive range of motion of the lower extremities are therefore considered descriptively (Table 5). A slight improvement in the passive range of motion of the measured degrees of freedom except for the mobility of the hip joint in lateral rotation (both sides) was observed. The improvements were not statistically significant.

**Table 5:** Overview of the results of the passive Range of Motion measurements

Passive Range of Motion	Degrees (T1)	Degrees (T2)
Hip		
Flexion (left) Flexion (right) Extension (left) Extension (right) Medial Rotation (left) Medial Rotation (right) Lateral Rotation (left) Lateral Rotation (right) Abduction (left) Abduction (right)	91.4 (± 5.5) 86.2 (± 10.8) 0.0 (± 5.5) -0.9 (± 7.6) 19.1 (± 9.7) 20.9 (± 12.6) 35.5 (± 22.3) 28.6 (± 15.7) 12.7 (± 7.9) 12.3 (± 7.5)	93.2 (± 13.3) 97.3 (± 13.7) 3.6 (± 6.0) 0.5 (± 6.9) 24.6 (± 16.2) 25.0 (± 9.5) 35.0 (± 20.5) 28.2 (± 15.5) 14.6 (± 6.1) 15.5 (± 7.9)
Knee		
Flexion (left) Flexion (right) Extension (left) Extension right)	134.1 (± 12.0) 136.8 (± 15.4) -11.8 (± 13.8) -9.6 (± 11.9)	135.0 (± 11.4) 137.3 (± 6.5) -6.8 (± 9.8) -7.3 (± 10.4)

T1: measurement pre−intervention; T2: measurement post−intervention; ±: standard deviation. n = 11

## Discussion

The results of this study show that the use of the HAL in an inpatient setting is feasible and safe. RAGT with the HAL gives adults with CP the possibility to explore their lower limb movement, somatosensory system, and posture without adverse events. Adult participants were motivated to work on their motor performance by using an exoskeleton as walking aid. RAGT with the HAL combined with an additional inpatient therapy concept consisting of physiotherapy, massage, medical exercise therapy and physician-performed manual medicine may have an influence on motor and gait parameters. None of these changes is statistically significant.

The current literature consists of studies with heterogeneous study protocols, a variety of dosage parameters, outcome measures, age ranges and different approaches to statistical analysis not including confounding variables. Thus, comparison of the results is difficult. The special feature of the HAL is that the participant are required to initiate movement themselves, they are not moved by the device only. No studies were found comparing the efficacy or energy expenditure of different types of RAGT or End-effector devices. Most studies examine children with CP (Lefmann et al., 2017). Only Lerner et al. (2017), Ueno et al. (2019) und Orekhov et al. (2020) included people with CP older than 18 years in their research. There has so far been no follow-up evaluation after HAL intervention, and there is no supporting evidence regarding the effects of frequency and duration of sessions or breaks between the sessions.

With the consensus of the motor plateau in people with CP in adolescence (Bottos et al., 2001; Jahnsen et al., 2004; Day et al., 2007; Hanna et al., 2009) point out that the number of subjects with CP who can walk decreases after the age of 18 years. As many as 34% of children who walk unsteadily and sometimes use a wheelchair appear to lose walking function as they age. However, Pirpiris et al., (2006) state that the strongest predictors of satisfaction in 90 children with CP are the ability to stand and perceived pain. Adults with CP experience disadvantages in social life and the labour market (Michelsen et al., 2006). About 40% of adult CP patients have a job only and less than 30% live independently (van Gorp et al., 2020). For adults with CP, it seems to be more important to develop the communication and technical skills required for the workplace so that they can communicate with and control their environment than to focus on small functional improvements (Moll & Cott, 2013). However, besides the mechanical characteristics affecting gait in patients with CP, factors such as cardiovascular endurance, cognitive abilities, and associated impairments such as visual limitations also influence patients’ capabilities (ACPR, 2013; Gage, 2009).

### Clinical Relevance

This study suggests the potential of RAGT with HAL for adults with CP to change walking parameters, such as 10MWT. Therefore, patients’ characteristics, training parameters and different neurological conditions and disorders need to be evaluated in further studies to get a clear idea of which group of patients is likely to benefit most from this therapy. Improvements of 10.3 s in the 10MWT (total) and 5 s in the 10MWT (max) seems to be clinically relevant in other populations. Changes in 6MWT results of 4.4 m do not reflect a clinically relevant change. An increase in the total score of the GMFM of 2.7% may present a clinically relevant change depending on the individual achievements. The assessment of the passive range of motion of the lower extremities also revealed positive changes. Since fast and clear motor progress in adults with CP is not to be expected, the time interval between the pre- and post-treatment assessments can be regarded as very short.

The use of the HAL is motivative and the activity required of the patient is an advantage for other devices. Because of interactive biofeedback, it is not possible for a patient to just consume the HAL therapy and pretend to be involved actively. Due to the high cost of RAGT, this form of therapy is available to very few patients. Independent use of the HAL at home is not possible. More standards need to be created under which patient-related and economic aspects of RAGT can be implemented. Much more research should also be done on the impact of the cardiovascular aspects of RAGT-supported exercise on adults with CP and their comorbidities.

From a qualitative point of view, it should be emphasized that RAGT supports participant’ movement not only passively, but also clearly assists and completes possible movements which are actively initiated by the participants. All participants felt motivated by the HAL to move more and enjoyed being part of this kind of robot-assisted therapy. The use of the HAL in patients with CP has many benefits such as: (i) creating a safe and motivating environment to explore walking abilities, (ii) supporting power and (iii) range of motion in highly repetitive walking training and increasing the safety in pre-walking motor abilities such as standing.

### Limitations

The present study has limitations. Due to the low sample size, the study is underpowered. Because the availability of the HAL was only temporary and patients’ concerns about COVID-19 infections, it was not possible to include more subjects in the study. The statistical model had to be adjusted and covariates had to be manually removed from the statistical model because of the insufficient degrees of freedom of the residuals. Additionally, no follow-up data were collected and therefore no medium- or long-term effect can be reported.

Due to the parallel application of RAGT sessions with physiotherapy, physician performed manual medicine, massage and exercise therapy, a clear performance bias must be pointed out. However, a control group without standard therapy or intervention could not be implemented for ethical reasons.

## Conclusion

The results indicate that RAGT with HAL can be performed safely in a hospital setting with adults with CP. Adults with CP show functional improvements after intensive active therapy with the HAL embedded in a participation-orientated therapy concept. However, these changes are not significant. The treatment approach with the HAL may be promising and needs to be evaluated under adjusted conditions such as larger sample sizes, longer intervention time for the single session as well for the intervention period. Much more qualitative research could provide further insights into the subjects’ progress and clinical outcomes. Further studies need to be conducted to establish a patient profile of the patient groups that benefit most from RAGT and specific training parameters.

## Data Availability

The data that support the findings of this study are available on request from the corresponding author. The data are not publicly available due to privacy or ethical restrictions.

## Abbreviations

10MWT: 10-metre Walking Test
6MWT: 6-minute Walking Test
AFO: Ankle-Foot Orthosis
AO: Ankle Orthosis
GMFCS: Gross Motor Function Classifications System
GMFM-88: Gross Motor Functions Measure (88 tasks)
HAL: Hybrid Assistive Limb
iBF: interactive Biofeedback
KMT: Klinik für Manuelle Therapie
MANCOVA: Multivariate Analysis of Covariance
pROM: Passive Range of Motion
RAGT: Robot-assisted gait training
SSW: Self-selected walking speed
T1: Day 1 (pre intervention assessment)
T2: Day 11 (post intervention assessment)
